# Tumor response and survival outcomes of salvage concurrent chemoradiotherapy with three-dimensional conformal radiotherapy and 5-fluorouracil/platinum-based chemotherapy for postoperative locoregional recurrence of esophageal squamous cell carcinoma

**DOI:** 10.1007/s10388-022-00936-3

**Published:** 2022-07-28

**Authors:** Renma Ito, Yoshiaki Nakamura, Hironori Sunakawa, Hisashi Fujiwara, Hidehiro Hojo, Naoki Nakamura, Takeo Fujita, Tomonori Yano, Hiroyuki Daiko, Tetsuo Akimoto, Takayuki Yoshino, Takashi Kojima

**Affiliations:** 1 Department of Internal Medicine, Komatsu Municipal Hospital, Komatsu, Japan; 2 Department of Gastroenterology and Gastrointestinal Oncology, National Cancer Center Hospital East, 6-5-1, Kashiwanoha, Kashiwa, Chiba, 277-8577 Japan; 3 Department of Gastroenterology and Endoscopy, National Cancer Center Hospital East, Chiba, Japan; 4 Division of Esophageal Surgery, National Cancer Center Hospital East, Chiba, Japan; 5 Division of Radiation Oncology, National Cancer Center Hospital East, Chiba, Japan; 6 Division of Radiology, St. Marianna University School of Medicine, Kawasaki, Japan; 7 Division of Esophageal Surgery, National Cancer Center Hospital, Tokyo, Japan

**Keywords:** Chemoradiotherapy, Esophageal cancer, Postoperative recurrence, Locoregional recurrence, Salvage therapy

## Abstract

**Background:**

Salvage concurrent chemoradiotherapy is effective against locoregional recurrence after curative resection of esophageal squamous cell carcinoma. However, there is no consensus on its application. We investigated the outcomes of salvage concurrent chemoradiotherapy (60 Gy in 30 fractions) with three-dimensional conformal radiotherapy and 5-fluorouracil/platinum-based chemotherapy.

**Methods:**

We retrospectively investigated the outcomes and prognostic factors in 51 patients with esophageal squamous cell carcinoma treated with salvage concurrent chemoradiotherapy.

**Results:**

The median follow-up was 17.5 (range, 2.8–116.1) months. The overall response, complete response, and partial response rates were 74.5%, 49.0%, and 25.5%, respectively. The median progression-free survival was 8.2 months; the 3-year progression-free survival rate was 22.9%. The median overall survival was 23.1 months; the 3-year overall survival rate was 40.7%. Overall survival was significantly longer in patients with a complete response than in those without (median overall survival: not reached *vs.* 15.3 months); 3-year overall survival rate: 62.5% *vs.* 20.3% (hazard ratio: 0.222; *P* < 0.001). Multivariate analysis showed that the independent prognostic factor for overall survival was < 25 mm longest diameter of metastatic lymph nodes (hazard ratio: 3.71).

**Conclusions:**

Salvage concurrent chemoradiotherapy (60 Gy in 30 fractions) with three-dimensional conformal radiotherapy and 5-fluorouracil/platinum-based chemotherapy was an effective and safe treatment for locoregional recurrence after curative resection of esophageal squamous cell carcinoma, especially in those approaching a complete response. Additionally, a shorter longest diameter of metastatic lymph nodes may be associated with better long-term survival.

**Supplementary Information:**

The online version contains supplementary material available at 10.1007/s10388-022-00936-3.

## Introduction

Esophageal cancer is the sixth leading cause of cancer-related deaths worldwide [1] and is histologically divided into squamous cell carcinoma and adenocarcinoma. Esophageal squamous cell carcinoma (ESCC) is common in East Asia and Africa and is more prevalent than esophageal adenocarcinoma in Europe and less prevalent than esophageal adenocarcinoma in North America [2]. Although survival outcomes in patients with ESCC have improved with the development of multidisciplinary treatment modalities [3, 4], postoperative recurrences still occur in 28–53% of patients who undergo curative resection [5, 6]. Locoregional recurrence is the most common type of recurrence. The use of salvage concurrent chemoradiotherapy (CCRT) for such recurrences after curative resection of ESCC has been reported to be more effective than surgery alone as salvage therapy, except for the treatment of cervical lymph node (LN) metastasis [7, 8]. However, since previous reports of salvage CCRT have included heterogeneous modalities, radiation doses, and concurrent chemotherapeutic regimens, the efficacy of a fixed method of salvage CCRT with a high radiation dose in three-dimensional conformal radiotherapy (3D-CRT) combined with 5-fluorouracil (5-FU)/platinum-based chemotherapy—accepted as one of the most effective approaches—is unknown [9–14].

In this study, we evaluated the efficacy of salvage CCRT (60 Gy in 30 fractions) with 3D-CRT and 5-FU/platinum-based chemotherapy for locoregional recurrence after curative resection of ESCC.

## Patients and methods

### Study design and patients

We retrospectively analyzed the outcomes of patients treated with 5-FU/platinum-based CCRT for locoregional recurrence after curative resection of ESCC at the National Cancer Center Hospital East (NCCHE). The correspondence of recurrence after curative resection of ESCC at NCCHE is given in Supplementary Fig. 1. The inclusion criteria were as follows: (1) pathologically proven ESCC; (2) locoregional recurrence defined as recurrence in anastomosis or a regional LN, including the supraclavicular and para-aortic LNs at the upper abdominal level, after curative resection (R0 radical esophagectomy with 2/3-field LN dissection) between April 2002 and December 2014; and (3) treated with salvage CCRT (60 Gy in 30 fractions) with 3D-CRT and 5-FU/platinum-based chemotherapy (cisplatin or nedaplatin). The exclusion criteria were as follows: (1) active cancer in other regions; (2) distant metastases; and (3) receiving radiotherapy and chemotherapy other than 5-FU/platinum-based regimens. Tumors were staged according to the American Joint Committee on Cancer/International Union Against Cancer tumor–node–metastasis (TNM) staging system (seventh edition) [15]. Although tumor response was primarily assessed according to the Response Evaluation Criteria in Solid Tumors (version 1.1) on computed tomography (CT) [16] and the modified criteria of the Japanese Society for Esophagus Diseases on endoscopy [17], for this study the definition of LN metastasis was > 10 mm size of the LN, and the definition of complete response (CR) was the disappearance of all visible lesions except scarred LNs. Adverse events (hematological and non-hematological toxicities of grade 3 or higher) and late adverse events were assessed using the Common Terminology Criteria for Adverse Events (version 4.0) [18].

The study was performed in accordance with the ethical principles based on the Declaration of Helsinki. The study design was approved by the Institutional Review Board of the National Cancer Center, Japan (approval number: 2017–120). Each patient provided written informed consent for diagnosis and treatment before the procedure was performed.

### Treatment and follow-up

External radiotherapy was administered using the 6- or 10-MV X-ray of a linear accelerator with a cumulative dose of 60 Gy (30 fractions at 2 Gy each). The gross tumor volume (GTV) was defined as a recurrence within anastomosis and/or one of the regional LNs. The clinical target volume (CTV) was determined with GTV plus 1 cm around GTV avoiding normal organs, and the planning target volume was defined as a 0.5–1.5-cm margin around the CTV to compensate for set-up variations and internal organ motion. 3D-CRT was used in all cases. CCRT consisted of 5-FU (700 mg/m^2^ on days 1–4 every 4 weeks) and cisplatin (70 mg/m^2^ on day 1 every 4 weeks) or 5-FU (800 mg/m^2^ on days 1–4 every 4 weeks) and nedaplatin (80 mg/m^2^ on days 1–4 every 4 weeks) for two cycles.

Tumor responses were assessed by CT and endoscopy after CCRT and were re-evaluated every 3–6 months for those who achieved a CR. Patients who achieved a partial response or stable disease were treated with an additional two cycles of the same chemotherapy until a CR was achieved or the disease progressed. Patients with disease progression received palliative chemotherapy or the best supportive care.

### Statistical analyses

We calculated the survival time from the start of salvage CCRT. We evaluated progression-free survival (PFS), defined as the time from the date of starting salvage CCRT to the date of disease progression or death, whichever came first, and overall survival (OS), defined as the time from the date of starting salvage CCRT to the date of death, using the Kaplan–Meier method, and analyzed PFS and OS according to the achievement of a CR. The log-rank test was used for the univariate analysis of the differences in the median OS. As in previous studies [13, 19, 20], we also analyzed OS according to sex, age (< 60 *vs.* ≥ 60 years), performance status (PS) of Eastern Cooperative Oncology Group (0 *vs.* 1/2), squamous cell carcinoma tumor marker (< 1.5 *vs.* ≥ 1.5 ng/mL), history of neoadjuvant or adjuvant chemotherapy, initial pathological stage (0/I/II *vs.* III/IV [TNM seventh edition]), recurrence interval (< 6.0 *vs.* ≥ 6.0 months), region of recurrence (single *vs.* multiple regions), longest metastatic LN diameter (< 25 *vs.* ≥ 25 mm), and chemotherapy regimen (5-FU plus cisplatin *vs.* 5-FU plus nedaplatin). Cox regression models were used for the multivariate analysis of the differences in the median OS; baseline variables with *P* < 0.10 in the univariate analysis were included in the multivariate analysis. All statistical analyses were conducted using EZR (Saitama Medical Center, Jichi Medical University, Saitama, Japan), a graphical user interface for version 3.6.3 of R (The R Foundation for Statistical Computing, Vienna, Austria). Statistical significance was defined as a two-tailed *P* < 0.05.

## Results

### Study flow and patient and tumor characteristics

The consort diagram is shown in Supplementary Fig. 2. Of the 959 patients with ESCC who underwent curative resection between April 2002 and December 2014, 230 patients (24.0%) had distant metastases, and 140 patients (14.6%) had locoregional recurrence only. Of the 140 patients with locoregional recurrence, 58 (41.4%) were treated with salvage CCRT. Seven patients (5.0%) were excluded from this study because they received chemotherapy other than 5-FU/platinum-based regimens or < 60 Gy of radiotherapy. The recurrence patterns of the 51 eligible patients were lymphnodal recurrences in 49 patients (96.7%), while 2 patients (3.3%) had anastomosis and lymphnodal recurrences. Of the 51 patients, 19 (29.4%) received a reduced dose or frequency of chemotherapy due to an underlying disease or chemotherapy toxicity. Radiotherapy was completed in all patients. Table 1 summarizes the patient and tumor characteristics. The median follow-up time was 17.5 (range, 2.8–116.1) months. The cohort predominantly included men (84.3%) with PS 0 (80.4%), a primary tumor located in the thorax (92.2%), and 5-FU plus cisplatin administered as the chemotherapy regimen for CCRT (80.4%).

**Table 1:** The patient and tumor characteristics

Patient characteristics	No. (%)
Patients	51
Age	
Median [range]	65 [45–76]
Gender	
Male	43 (84.3)
ECOG PS	
0	41 (80.4)
1	9 (17.6)
2	1 (2.0)
Tumor marker (SCC)	
ng/mL, median[range]	1.3 [0.2–5.4]
Location of primary tumor	
Cervix	3 (5.9%)
Upper thorax	5 (9.8%)
Middle thorax	28 (54.9%)
Lower thorax	14 (27.5%)
Abdominal	1 (2.0%)
Neoadjuvant or adjuvant chemotherapy	
Yes	36 (70.6%)
Neoadjuvant	28 (54.9)
Adjuvant	8 (15.7%)
Regimen of neoadjuvant or adjuvant chemotherapy	
DCF	12 (33.3%)
FP	22 (61.1%)
FN	2 (5.6%)
Initial UICC pStage	
0	2 (3.9%)
I	3 (5.9%)
II	12 (23.5%)
III	31 (60.8%)
IV	3 (5.9%)
Histology	
Well differentiated	7 (13.7%)
Moderately differentiated	16 (31.4%)
Poorly differentiated	27 (52.9%)
Basaloid	1 (2.0%)
Interval to recurrence	
Month, median [range]	10 [2–80]
Recurrence site	
Supraclavicular region LN	8 (15.7%)
Mediastinal LN	32 (62.7%)
Abdominal LN	3 (5.9%)
Multiple LN	8 (15.7%)
Anastomosis	2 (3.9%)
Number of recurrent LN	
1	31 (60.8%)
≥ 2	20 (39.2%)
Longest diameter of recurrent LN	
mm, median [range]	18 [12–38]
Chemotherapy regimen of CCRT	
FP	41 (80.4%)
FN	10 (19.6%)

*ECOG* Eastern Cooperative Oncology Group, *DCF* Docetaxel + Cisplatin + 5FU, *FP* 5FU + Cisplatin, *FN* 5FU + Nedaplatin, *LN* Lymph node, Concurrent chemoradiotherapy

### Treatment outcomes

**Fig. 1:**
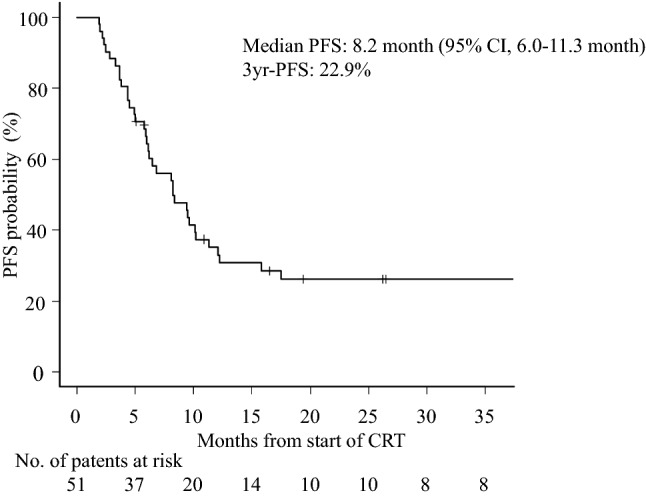
Kaplan–Meier estimates of progression-free survival

**Fig. 2:**
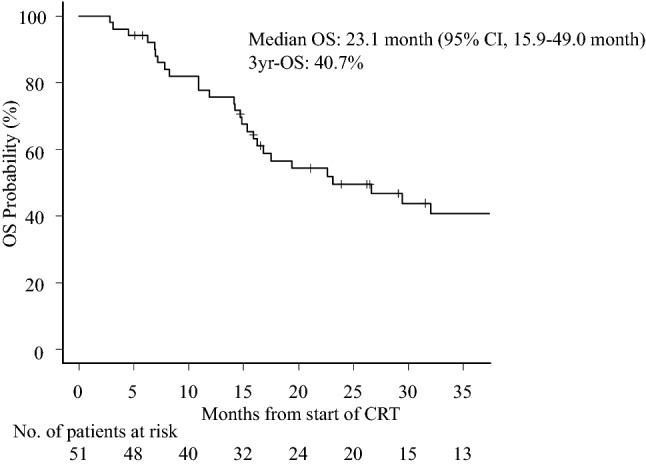
Kaplan–Meier estimates of overall survival

**Fig. 3:**
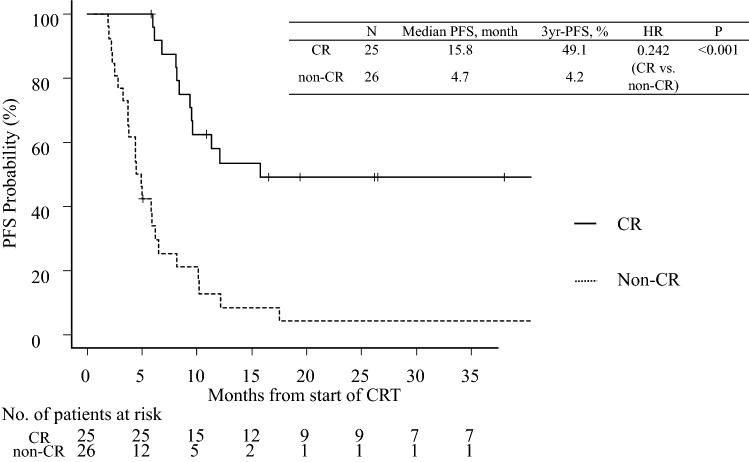
Kaplan–Meier estimates of progression-free survival according to response

**Fig. 4:**
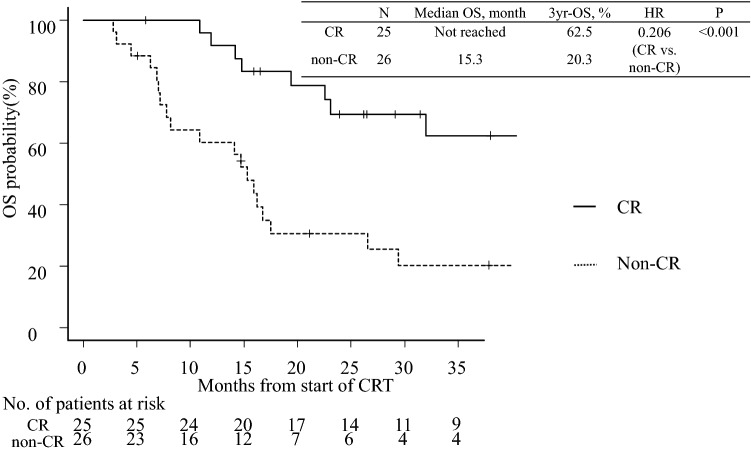
Kaplan–Meier estimates of overall survival according to response

The overall response rate was 74.5% (38/51), with a CR rate of 49.0% (25/51) (Table 2). The median PFS was 8.2 (95% confidence interval [CI] 5.8–10.2) months; the 3-year PFS rate was 22.9% (Fig. 1). The median OS was 23.1 (95% CI 15.9–49.0) months; the 3-year OS rate was 40.7% (Fig. 2). The median PFS was significantly longer in patients with a CR than in those without (15.8 *vs.* 4.7 [95% CI 9.4–N/A *vs.* 3.7–6.2] months); the 3-year PFS rate was 49.1% *vs.* 4.2% (hazard ratio [HR]: 0.242 [95% CI 0.051–0.432]; *P* < 0.001) (Fig. 3). The median OS was also significantly longer in patients with a CR than in those without (not reached *vs.* 15.3 [95% CI 23.1–N/A *vs.* 7.8–17.5] months); the 3-year OS rate was 62.5% *vs.* 20.3% (HR: 0.206 [95% CI 0.028–0.440]; *P* < 0.001) (Fig. 4).

**Table 2:** Treatment response

Treatment response	No. (%)
Complete response (CR)	25 (49.0%)
Partial response (PR)	13 (25.5%)
Stable disease (SD)	8 (15.7%)
Progression of disease (PD)	5 (9.8%)

### Toxicity

Treatment-related adverse events of grade 3 or higher and late adverse events are shown in Table 3. Adverse events of grade 3 or higher were all acute adverse events and no grade 5 toxicities were observed. Grade 4 Leukopenia, neutropenia and thrombocytopenia were observed in two patients (3.9%) each. Grade 3 fatigue was observed in nine patients (17.6%), grade 3 leukopenia in eight patents (15.6%), grade 3 neutropenia in seven patients (13.7%), grade 3 anemia and nausea in four patients (7.8%) each, grade 3 hyponatremia in two patients (3.9%), and grade 3 thrombocytopenia, febrile neutropenia, hyperglycemia, and pneumonia in one patient (2.0%). Among the late adverse events, grade 1 radiation pneumonia was observed in five patients (9.8%), and grade 1 pleural effusion was noted in one patient (2.0%).

**Table 3:** Adverse events

	No. (%)	
	Gr3	Gr4
*Adverse events (hematological and non-hematological toxicities of grade 3 or higher)*		
Leukopenia	8 (15.6%)	2 (3.9%)
Neutropenia	7 (13.7%)	2 (3.9%)
Anemia	4 (7.8%)	0 (0%)
Thrombocytopenia	1 (2.0%)	2 (3.9%)
Febrile neutropenia	1 (2.0%)	0 (0%)
Hyponatremia	2 (3.9%)	0 (0%)
Hyperglycemia	1 (2.0%)	0 (0%)
Fatigue	9 (17.6%)	0 (0%)
Nausea	4 (7.8%)	0 (0%)
Pneumonia	1 (2.0%)	0 (0%)

	No. (%)	
	Gr1	≤ Gr2
*Late adverse events*		
Radiation pneumonia	5 (9.8%)	0 (0%)
Pleural effusion	1 (2.0%)	0 (0%)

### Association of tumor response with survival

In univariate analysis, PS 0 and the longest metastatic LN diameter of < 25 mm were associated with significantly better OS (*P* = 0.009 and 0.001, respectively). Multivariate analysis demonstrated that the independent prognostic factor for OS was the longest metastatic LN diameter of < 25 mm (HR, 3.71; 95% CI, 1.52–9.05) (Table 4).

**Table 4:** Univariate analyses and multivariate analyses of OS

Factor	Group	*N*	Median OS, months	Univariate analysis	Multivariate analysis	
				*P* value	Hazard ratio [95%CI]	*P* value
Sex	Male	43	22.6	0.415	–	–
	Female	8	26.6			
Age	< 60 years old	10	21.0	0.076	0.56 [0.22–1.38]	0.209
	≥ 60 years old	41	42.0			
PS	0	41	32.0	0.009	2.32 [0.91–5.91]	0.077
	1/2	10	14.2			
SCC at recurrence	< 1.5 ng/ml	33	42.0	0.054	1.50 [0.71–3.20]	0.284
	≥ 1.5 ng/ml	18	14.2			
Neoadjuvant or adjuvant chemotherapy	No	15	19.4	0.432	–	–
	Yes	36	29.4			
pStage	0/I/II	17	42.0	0.236	–	–
	III/IV	34	17.5			
Interval between surgery and recurrence	< 6.0 month	18	15.9	0.142		
	≥ 6.0 month	33	32.0			
Number of recurrence node	Single	31	19.4	0.958	–	–
	Multiple	20	29.4			
Region of recurrence	Single region	42	22.6	0.334	–	–
	Multiple region	9	32.0			
Longest diameter of metastatic LN	< 25 mm	41	29.4	0.001	3.71 [1.52–9.05]	0.003
	≥ 25 mm	10	7.0			
Regimen	FP	41	26.6	0.454	–	–
	FN	10	15.4			

*FP* 5FU + Cisplatin, *FN* 5FU + Nedaplatin

## Discussion

In previous studies, high radiation dose, and 3D-CRT or 5-FU/platinum-based chemotherapy are reported to be effective for salvage CCRT for the treatment of locoregional recurrence after curative resection of ESCC [9–14]. This study reported the treatment efficacy of salvage CCRT with a fixed approach at a dose of 60 Gy in 30 fractions of 3D-CRT combined with 5-FU/platinum-based chemotherapy. The median OS was approximately 2 years, and approximately half of the patients achieved a CR, which was associated with longer survival.

The methods involving radiotherapy and chemotherapy for salvage CCRT for locoregional recurrence of ESCC were inconsistent in previous reports (> 20 cases) [9–11, 13, 14, 19, 21–23], and the overall response rate was > 70% (Supplementary Table 1). However, the 3-year survival rate ranged from 10.5% to 51.8%, with a median OS ranging between 13 and 43 months. More recent reports tended towards better survival outcomes. Our results, with a 3-year OS rate of 40.7% and a median OS of 23.1 months, were consistent with the recent reports.

Compared to two-dimensional radiotherapy, 3D-CRT has improved anatomical imaging, significantly enhancing target delineation and sparing neighboring tissues by optimizing the dose distribution. Thus, 3D-CRT is expected to reduce radiation-induced side effects [24]. Late toxicity events were previously thought to be mainly attributed to radiation-induced side effects. This study reported that 9.8% of the patients showed grade 1 toxicity and reported no toxicities that were grade 3 or more. However, previous reports have shown 2.4%–3.3% of patients with grade 3 toxicity [10, 14] and 4.3%–11.4% with grade 1 or 2 toxicity [11, 14, 21]. Therefore, we assumed that 3D-CRT reduced the radiation-induced side effects despite the high-dose radiation reported in this study. Additionally, late toxicity events in this study using 60 Gy were lower than that of a definitive CCRT study for esophageal cancer using 50.4 Gy regimen [25]. This might be due to differences in target lesion and irradiated area.

Grade 3/4 toxicity of salvage CCRT (60 Gy in 30 fractions of 3D-CRT combined with 5-FU/platinum-based chemotherapy) for locoregional recurrence of ESCC reported in this study was observed in < 15% of patients with hematological toxicities and < 20% of patients with non-hematological toxicities. However, in previous studies, it was observed in 17.4%–36.7% of patients with hematological toxicities and 14.0%–33.3% of patients with non-hematological toxicities [13, 19, 21–23]. Therefore, we thought that it did not differ from previously reported studies involving lower-dose radiotherapy or other chemotherapy regimens. Consequently, high-dose salvage CCRT (60 Gy in 30 fractions of 3D-CRT combined with 5-FU/platinum-based chemotherapy) for locoregional recurrence after curative resection of ESCC was well-tolerated.

In previous studies, the prognostic factors for locoregional recurrence after curative resection of ESCC were 3D-CRT, a radiation dose of ≥ 60 Gy, chemotherapy regimens, the time from surgery to recurrence, the size (diameter) of LN metastases, number of LN metastases, and location of LN metastases [12, 13, 19, 22, 23, 26–28]. In our study, there was no difference in radiation therapy because all cases received 60 Gy radiation doses in 3D-CRT. Additionally, all cases received radiotherapy with 5-FU and platinum-based chemotherapy. Also, there were no significant differences in the prognostic factors between the patients receiving the regimens that used cisplatin and nedaplatin in this study. Besides chemotherapy and the method of radiation therapy, the examination of prognostic factors revealed that a shorter longest diameter of metastatic LNs was a good prognostic factor.

We reported high 3-year PFS and OS rates in patients owing to the therapeutic effect of CR. Therefore, reaching CR was deemed necessary for long-term survival. The favorable prognosis in the CR cases of salvage CCRT makes it worthwhile considering a treatment regimen that enhances the treatment response. Bao et al*.* [23] reported a better prognosis and response rate with a docetaxel and cisplatin regimen than with a 5-FU and cisplatin regimen as salvage CCRT. Additionally, Tamaki et al*.* [29] reported that a combination of docetaxel, cisplatin, and 5-FU had better outcomes than 5-FU and cisplatin in advanced esophageal cancer, with acceptable toxicity profiles. Furthermore, Antonia et al*.* [30] reported improved therapy when an immune checkpoint inhibitor was added after chemoradiotherapy in patients with lung cancer. Therefore, taxane and platinum-based chemotherapy, 5-FU plus platinum and taxane-based chemotherapy, and the addition of an immune checkpoint inhibitor after chemoradiotherapy in salvage CCRT may be worth considering in the future.

This study has some limitations. First, this is a retrospective, single-center study. Second, this study included patients who received perioperative chemotherapy. However, there was no effect of combination chemotherapy on prognosis in multivariate analysis.

In conclusion, salvage CCRT (60 Gy in 30 fractions of 3D-CRT in combination with 5-FU/platinum-based chemotherapy) for locoregional recurrence after curative resection of ESCC was an effective and safe treatment. Approximately 40% of the cases achieved long-term survival following salvage CCRT, particularly in cases with CR. In addition, cases with a shorter longest diameter of metastatic LNs may be associated with improved long-term survival after salvage CCRT.

## Supplementary Information

Below is the link to the electronic supplementary material.

Supplementary file1 (PPTX 35 KB)

Supplementary file2 (PPTX 48 KB)
