# Supplementary material for: Tumor response and survival outcomes of salvage concurrent chemoradiotherapy with three-dimensional conformal radiotherapy and 5-fluorouracil/platinum-based chemotherapy for postoperative locoregional recurrence of esophageal squamous cell carcinoma
Source: Esophagus. 2022 Jul 28;19(4):645–52. doi: 10.1007/s10388-022-00936-3 (PMC9436848; doi:10.1007/s10388-022-00936-3)
Supplement: Supplementary file 1 — Supplementary file1 (PPTX 35 KB) [file 10388_2022_936_MOESM1_ESM.pptx]

## Slide 1
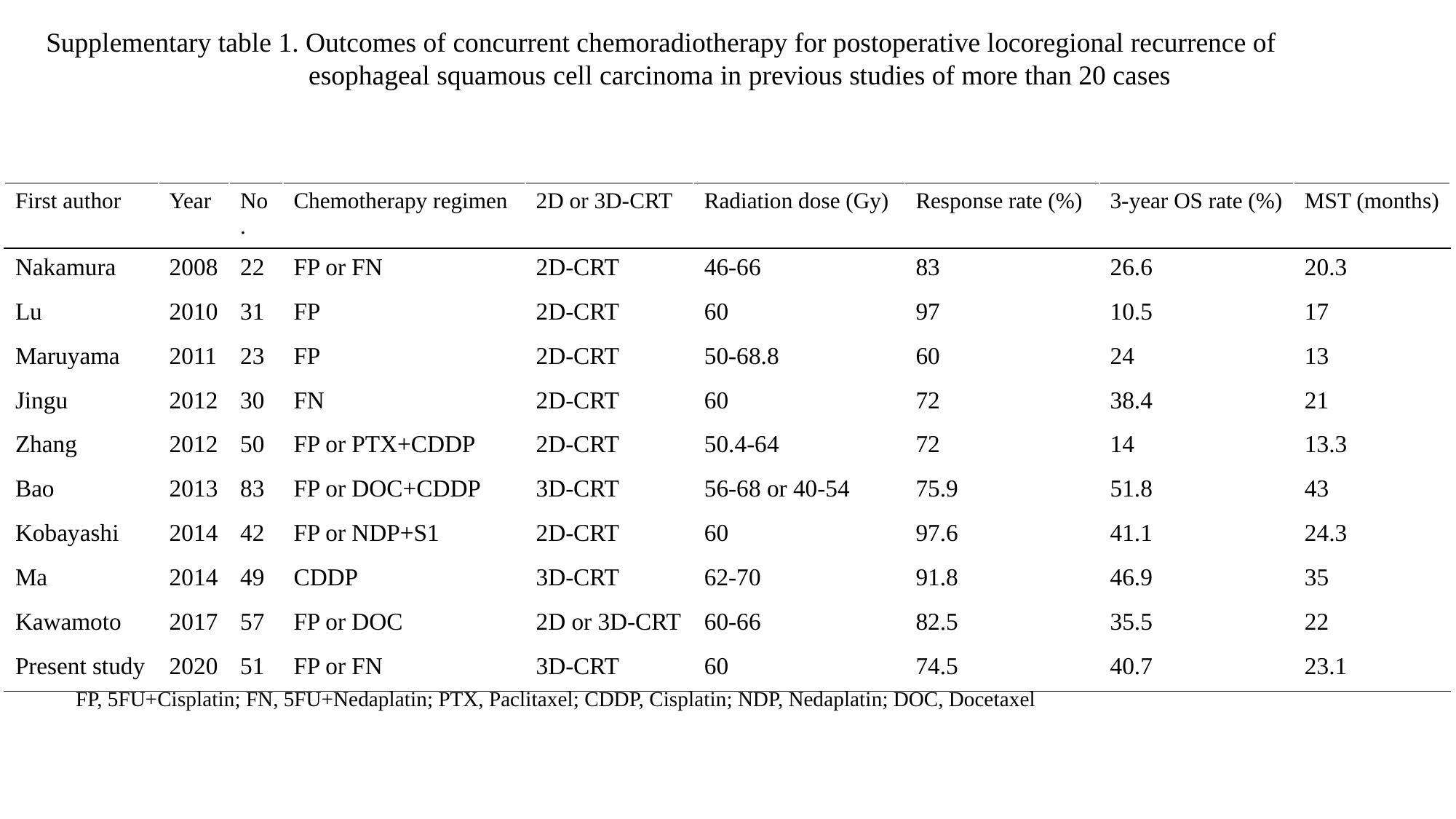

Supplementary table 1. Outcomes of concurrent chemoradiotherapy for postoperative locoregional recurrence of
 esophageal squamous cell carcinoma in previous studies of more than 20 cases
| First author | Year | No. | Chemotherapy regimen | 2D or 3D-CRT | Radiation dose (Gy) | Response rate (%) | 3-year OS rate (%) | MST (months) |
| --- | --- | --- | --- | --- | --- | --- | --- | --- |
| Nakamura | 2008 | 22 | FP or FN | 2D-CRT | 46-66 | 83 | 26.6 | 20.3 |
| Lu | 2010 | 31 | FP | 2D-CRT | 60 | 97 | 10.5 | 17 |
| Maruyama | 2011 | 23 | FP | 2D-CRT | 50-68.8 | 60 | 24 | 13 |
| Jingu | 2012 | 30 | FN | 2D-CRT | 60 | 72 | 38.4 | 21 |
| Zhang | 2012 | 50 | FP or PTX+CDDP | 2D-CRT | 50.4-64 | 72 | 14 | 13.3 |
| Bao | 2013 | 83 | FP or DOC+CDDP | 3D-CRT | 56-68 or 40-54 | 75.9 | 51.8 | 43 |
| Kobayashi | 2014 | 42 | FP or NDP+S1 | 2D-CRT | 60 | 97.6 | 41.1 | 24.3 |
| Ma | 2014 | 49 | CDDP | 3D-CRT | 62-70 | 91.8 | 46.9 | 35 |
| Kawamoto | 2017 | 57 | FP or DOC | 2D or 3D-CRT | 60-66 | 82.5 | 35.5 | 22 |
| Present study | 2020 | 51 | FP or FN | 3D-CRT | 60 | 74.5 | 40.7 | 23.1 |
FP, 5FU+Cisplatin; FN, 5FU+Nedaplatin; PTX, Paclitaxel; CDDP, Cisplatin; NDP, Nedaplatin; DOC, Docetaxel
