# Supplementary material for: Tumor response and survival outcomes of salvage concurrent chemoradiotherapy with three-dimensional conformal radiotherapy and 5-fluorouracil/platinum-based chemotherapy for postoperative locoregional recurrence of esophageal squamous cell carcinoma
Source: Esophagus. 2022 Jul 28;19(4):645–52. doi: 10.1007/s10388-022-00936-3 (PMC9436848; doi:10.1007/s10388-022-00936-3)
Supplement: Supplementary file 2 — Supplementary file2 (PPTX 48 KB) [file 10388_2022_936_MOESM2_ESM.pptx]

## Slide 1
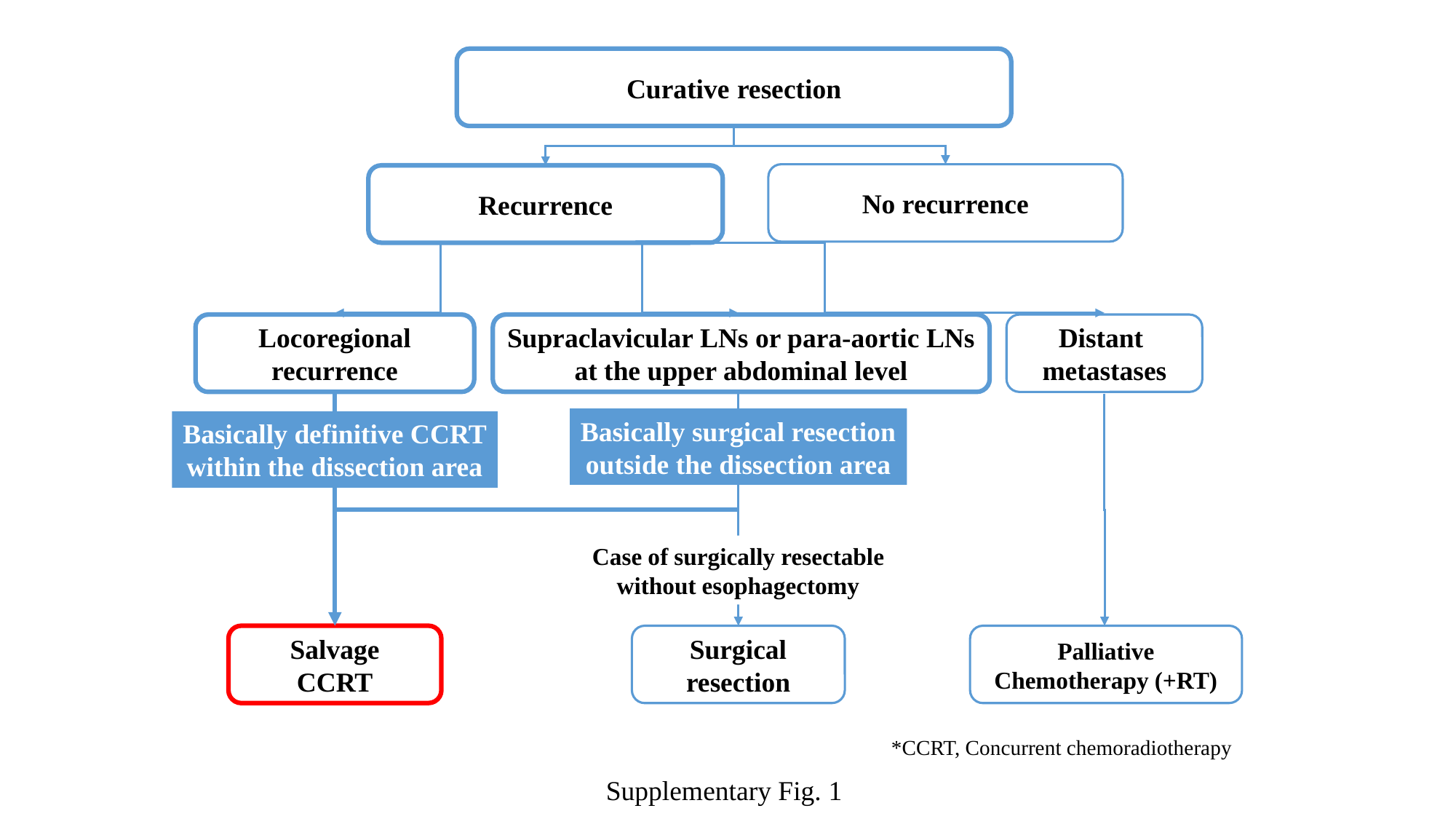

Curative resection
No recurrence
Recurrence
Locoregional recurrence
Supraclavicular LNs or para-aortic LNs at the upper abdominal level
Distant
metastases
Basically surgical resection
outside the dissection area
Basically definitive CCRT
within the dissection area
Case of surgically resectable
without esophagectomy
Salvage
CCRT
Surgical resection
Palliative
Chemotherapy (+RT)
*CCRT, Concurrent chemoradiotherapy
Supplementary Fig. 1

## Slide 2
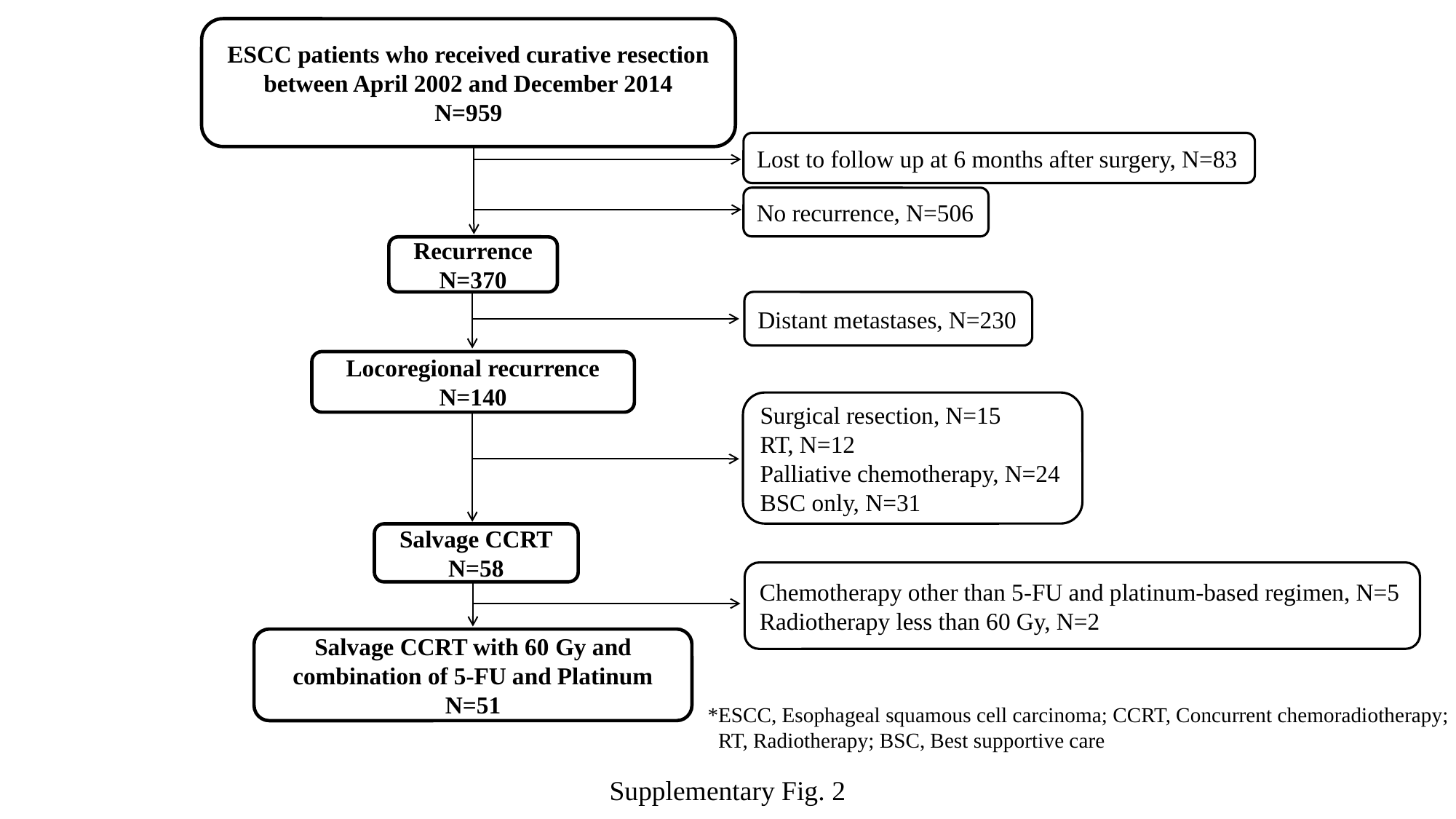

ESCC patients who received curative resection between April 2002 and December 2014
N=959
Lost to follow up at 6 months after surgery, N=83
No recurrence, N=506
Recurrence
N=370
Distant metastases, N=230
Locoregional recurrence
N=140
Surgical resection, N=15
RT, N=12
Palliative chemotherapy, N=24
BSC only, N=31
Salvage CCRT
N=58
Chemotherapy other than 5-FU and platinum-based regimen, N=5
Radiotherapy less than 60 Gy, N=2
Salvage CCRT with 60 Gy and combination of 5-FU and Platinum
N=51
*ESCC, Esophageal squamous cell carcinoma; CCRT, Concurrent chemoradiotherapy;
 RT, Radiotherapy; BSC, Best supportive care
Supplementary Fig. 2
